# ENaC inhibitors for the management of lithium related polyuria: a systematic review

**DOI:** 10.1192/j.eurpsy.2026.10185

**Published:** 2026-07-31

**Authors:** R. Strawbridge, M. Ott, U. Werneke, M. Abbott, A. Prabhu, A. H. Young, J. M. Meyer

**Affiliations:** 1 Institute of Psychiatry, Psychology & Neuroscience, King’s College London, London, United Kingdom; 2 Department of Public Health and Clinical Medicine, Nephrology; 3 Department of Clinical Sciences, Psychiatry, Sunderby Research Unit, Umea University, Umea, Sweden; 4 Guy’s and St Thomas’s NHS Foundation Trust, London, United Kingdom; 5 Department of Psychiatry, University of California, San Diego School of Medicine, California, United States

## Abstract

**Introduction:**

By entering collecting duct principal cells via the epithelial sodium channel (ENaC), lithium is capable of inducing vasopressin insensitivity, resulting in excessive urine production, nephrogenic diabetes insipidus (NDI) and potential for other long-term forms of renal dysfunction. ENaC inhibitors (ENaC-I) such as amiloride have been shown in animal models to minimise this adverse effect, and while ENaC-I are often considered an effective strategy, the literature on ENaC-I for lithium-related polyuria has not yet been synthesised despite the importance of this topic.

**Objectives:**

This review aimed to identify all published evidence for adjunctive use of an ENaC-I for lithium-related polyuria to estimate its effectiveness while also exploring potential moderators of effectiveness.

**Methods:**

The systematic search covered databases MEDLINE, EMBASE and PsycINFO complemented by handsearches, aiming to identify all studies of ENaC-I interventions in lithium-treated patients with pre- and post-ENaC-I polyuria as outcomes.

**Results:**

10 studies totalling 25 participants were eligible for inclusion and were synthesised narratively. Amiloride was the ENaC-I used in 24/25 participants, and triamterene in the other. 8/10 publications were single case reports, 4 of which presented substantial confounding issues. Clear improvements to polyuria were demonstrated in most papers, including the two larger studies.

**Conclusions:**

Although it appears very likely that ENaC inhibitors help ameliorate polyuria in lithium-treated patients, the quantity and quality of evidence is low. Heterogeneity in patient characteristics, intervention characteristics and study designs limit conclusions regarding the contribution of factors likely to influence ENaC-I effectiveness for lithium-induced polyuria. Besides, adverse effects require further exploration.

**Disclosure of Interest:**

None Declared

